# An Initiative for the Early Detection and Intervention of Postoperative Lymphedema in Patients With Breast Cancer by Occupational and Physical Therapists

**DOI:** 10.7759/cureus.91857

**Published:** 2025-09-08

**Authors:** Yoshiteru Akezaki, Eiji Nakata, Masato Kikuuchi, Ritsuko Tominaga, Hideaki Kurokawa, Kenjiro Aogi, Shinsuke Sugihara

**Affiliations:** 1 Division of Physical Therapy, Kochi Professional University of Rehabilitation, Kochi, JPN; 2 Department of Orthopaedic Surgery, Okayama University Hospital, Okayama, JPN; 3 Department of Rehabilitation Medicine, National Hospital Organization Shikoku Cancer Center, Ehime, JPN; 4 Department of Breast Oncology, National Hospital Organization Shikoku Cancer Center, Ehime, JPN

**Keywords:** breast cancer, early detection, early intervention, lymphedema, occupational therapy, physical therapy, preventive care, rehabilitation, surgery

## Abstract

Background

Early detection and treatment of lymphedema is essential because once symptoms develop, it is a lifelong condition that is increasingly difficult to manage as the disease progresses. This study examined the effectiveness of an initiative whereby occupational and physical therapists identified possible lymphedema cases early and referred patients to physicians for intervention.

Methods

The subjects of this study were 107 consecutive breast cancer patients who underwent axillary lymph node dissection at our hospital between April 2020 and June 2021, and received rehabilitation intervention postoperatively. Occupational and physical therapists evaluated the patient’s upper extremity function postoperatively and recommended that a physician examine patients with suspected lymphedema. Thereafter, a doctor examined the patient, and a nurse provided lymphedema education, including guidance on activities of daily living, skincare, and lymphatic drainage to prevent the lymphedema from worsening. Effectiveness was based on upper extremity circumference and lymphedema staging.

Results

We were able to refer 33 patients with suspected lymphedema to a doctor. These patients received medical attention and early intervention by nurses. There was no significant improvement in the upper extremity circumference due to the outpatient intervention, but no worsening either.

Conclusions

Postoperative breast cancer patients are at risk of developing lymphedema after discharge from the hospital. Postoperative assessment by occupational and physical therapists led to the detection of lymphedema at an early stage. As a result, patients were able to prevent the lymphedema from worsening through early treatment, and this intervention method was found to be effective to a certain degree. Therefore, this method is a useful system for identifying and managing lymphedema.

## Introduction

Breast cancer is the fourth most common cause of death worldwide, the leading cause of death among females, and its global burden is on the rise [1]. Advances in diagnosis and treatment techniques have improved the five-year survival rate for breast cancer patients [2-4], as well as reduced mortality and increased overall survival rates [5-8]. Breast cancer treatment options include surgery, chemotherapy, radiation therapy, and hormone therapy. Patients are offered their choice of treatments based on their characteristics and disease status.

Radical treatment requires surgery, and in many cases, surgery is the first treatment option [9]. Side effects after treatment for breast cancer include pain [10], upper extremity dysfunction [11], axillary web syndrome [12], and lymphedema [10]. Breast cancer-related lymphedema is a common and problematic complication after surgery [13]. It is characterized by swelling of the arms due to the accumulation of lymphatic fluid in the interstitial space, which does not return to the bloodstream [14-16]. Lymphedema is a potential side effect that can develop at any time, even more than a year after surgery [17]. If left untreated, it causes severe symptoms, including impaired physical function, decreased physical activity, depression, emotional distress, and decreased quality of life. It can also cause cellulitis and lymphangitis, and prolonged lymphedema can lead to lymphangiosarcoma [18].

Once symptoms develop, lymphedema can be a life-long condition that is increasingly difficult to manage as the disease progresses. However, symptoms can be controlled if detected and treated before they progress significantly [19]. Therefore, early detection and treatment are essential. Although lymphedema benefits from early intervention, access to lymphedema-specific services for screening and diagnosis is not well established [20,21]. Early lymphedema detection requires patients to identify it for themselves, but there are limits to what can be expected of patients with inadequate medical knowledge [22]. We hypothesized that establishing a system of regular evaluation and diagnosis by medical professionals would promote early detection and treatment of the condition.

In this study, we examined the effectiveness of an initiative that enables occupational and physical therapists to detect and intervene in cases with lymphedema by referring patients with possible lymphedema to physicians at an early stage.

## Materials and methods

Study design

This was a retrospective study based on medical records.

Patient population

The subjects of this study were 107 consecutive breast cancer patients who underwent axillary lymph node dissection at our hospital between April 2020 and June 2021 and received rehabilitation intervention postoperatively. This study included all the patients. Exclusion criteria included patients who were difficult to evaluate postoperatively. We conducted this study in accordance with the ethical standards of the Shikoku Cancer Center Ethics Committee, which approved this study (Approval No. 2018-45).

Methods

Occupational and physical therapists evaluated the upper extremity function of the patients postoperatively as a screening for lymphedema and recommended that patients with suspected lymphedema be examined by a physician, who then performed the examination.

Screening for Lymphedema

After the patient was discharged from the hospital, occupational and physical therapists evaluated the patient during postoperative visits to the hospital for treatment, which included measurements of upper extremity circumferences. Evaluations were performed each time the patient visited the hospital. The timing of the visits was every three or four weeks for chemotherapy, every four weeks for radiation therapy, and every three months for hormone therapy. Patients with no treatment were scheduled between six months and one year after surgery. The suspected lymphedema evaluation was as follows: 1. The circumferences of the upper limbs had increased by 1.5-2.0 cm or more compared with before surgery, and firmness of the arm when palpated was present; 2. There were no obvious signs of lymphedema at the time, but the patient was very concerned about lymphedema and wanted to receive education about lymphedema.

The circumferences of the upper limb were measured at the dorsum manus and wrist, as well as 5-cm distally and 10-cm proximally from the elbow fossa line. The circumference of the upper extremity was measured by an occupational therapist, who was aware of whether the patient had undergone outpatient intervention. Occupational and physical therapists also provided lifestyle guidance based on the evaluation results.

Outpatient Intervention

Following the suspected lymphedema identification, a doctor examined the patient, and a nurse provided education on lymphedema management. Education included guidance on activities of daily living (ADL), skincare, and lymphatic drainage to prevent the condition from worsening. In patients for whom outpatient intervention alone was clearly insufficient to maintain or improve lymphedema, the physician decided to admit them for intensive drainage or lymphovenous anastomosis.

Additional Measurements

The survey items regarding lymphedema stage and upper limb circumferences, which were measured at the time of outpatient intervention, were recorded again after three months and one year. A physician assessed the clinical stage of upper extremity lymphedema as per the International Society of Lymphology (ISL) criteria [23]. Similar to the screening assessments, physicians measured the circumferences of the upper limb at the dorsum manus and wrist, as well as 5-cm distally and 10-cm proximally from the elbow fossa line.

Statistical analysis

Calculation of sample size was performed using G*Power (version 3.1.9.4, Heinrich-Heine-Universität Düsseldorf, Düsseldorf, Germany), with alpha=0.05 and power=0.9. The number of patients was set at 107, foreseeing a sample loss of around 20%.

Upper limb circumferences were compared over time using a repeated measures analysis of variance (ANOVA) with Tukey’s post-hoc tests. The statistical analysis was performed with IBM SPSS Statistics for Windows, Version 29 (Released 2022; IBM Corp., Armonk, New York, United States). P-values of <0.05 were considered statistically significant.

## Results

Patient status

**Figure 1:**
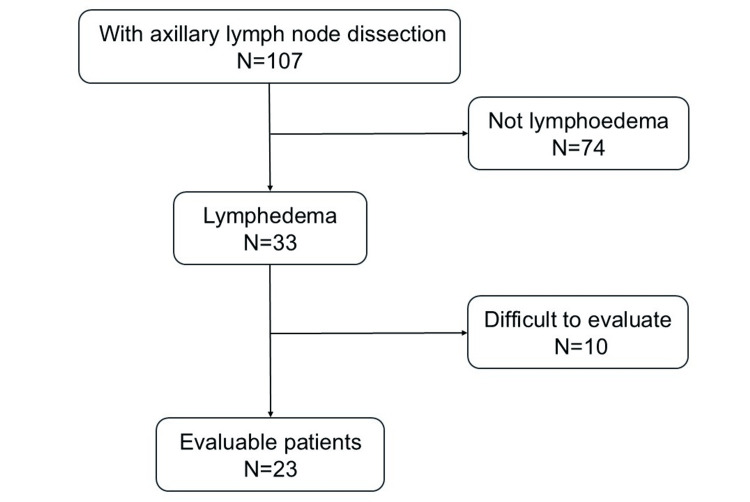
Flowchart of the study participants

The flowchart of the patients in the study is shown in Figure 1.

The characteristics of the patients are shown in Table 1.

**Table 1:** Patient characteristics ^a^Mean ± Standard deviation

Variables	
Sex: Female/Male (n)	23/0
Age (years)^a^	57.2±9.8
Body mass index (kg/m^2^)^a^	22.6±3.0
Lymph node dissection:Ⅰ/Ⅱ/Ⅲ (n)	19/3/1
Number of days from surgery to first outpatient start date (days)^a^	213.9±147.3
Marital status: Yes/No (n)	20/3
Number of people living together (n)^a^	2.4±1.3

Of the 107 patients, 33 were evaluated for suspected lymphedema by occupational and physical therapists. A total of 23 patients were evaluated during the intervention. Of the 23 patients, two required hospitalization for intensive drainage, and three received lymphaticovenous anastomosis (LVA).

Changes in upper extremity circumference

The changes in the upper limb circumference are shown in Table 2.

**Table 2:** Changes in the upper limb circumference Data reported are the mean ± standard deviation. ^a^Intervention vs 3 months; ^b^3 months vs 1 year; ^c^Intervention vs 1 year

Variables	Intervention (n=23)	3 months (n=23)	1 year (n=23)	DF	F-value	EF	p-value^a^	p-value^b^	p-value^c^
Operative side									
10 cm proximal from the elbow fossa line (cm)	25.5±3.2	25.5±3.2	25.5±3.3	2	0.014	0.00	0.984	0.996	0.996
5 cm distal from the elbow fossa line (cm)	22.7±2.0	22.6±2.2	22.7±2.2	2	0.027	0.00	0.971	0.991	0.994
Wrist (cm)	14.8±0.7	14.9±0.9	14.8±0.9	2	0.266	0.01	0.790	0.818	0.999
Dorsum manus (cm)	17.8±0.9	17.9±1.0	17.8±0.9	2	0.318	0.01	0.724	0.838	0.978
Non-operative side									
10 cm proximal from the elbow fossa line (cm)	25.0±3.4	24.9±3.3	24.9±3.4	2	0.111	0.1	0.929	0.997	0.899
5 cm distal from the elbow fossa line (cm)	22.5±2.2	22.2±2.2	22.1±2.1	2	2.329	0.1	0.210	0.960	0.960
Wrist (cm)	14.9±1.0	15.0±1.0	14.8±1.0	2	0.690	0.03	0.859	0.476	0.795
Dorsum manus (cm)	17.4±0.9	17.5±1.1	17.4±0.9	2	0.740	0.03	0.844	0.451	0.788

The circumference passing through the second to fifth metacarpophalangeal joints, the dorsum manus, wrist, as well as 5-cm distally and 10-cm proximally from the elbow fossa line showed no significant changes between the outpatient intervention, three months later, or one year later.

Lymphedema staging

**Figure 2:**
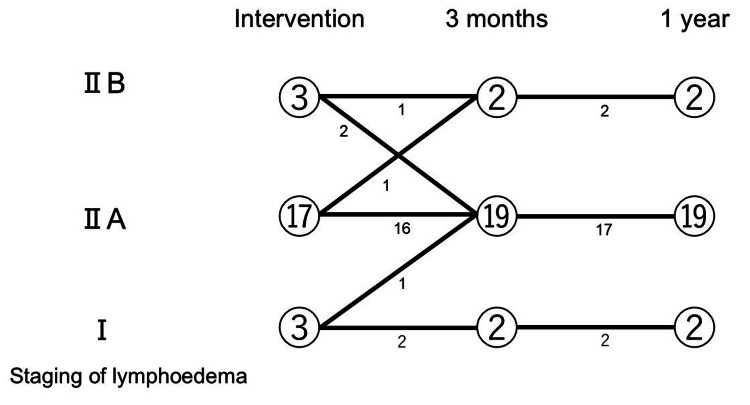
Changes in lymphedema staging

The changes in lymphedema staging are shown in Figure 2.

As for the lymphedema staging, two patients were deteriorating one year after the outpatient program intervention. One changed from I to IIA, and one from IIA to IIB one year after the outpatient intervention. Two patients improved, both from IIB at the time of the outpatient intervention to IIA one year later. Nineteen patients showed no changes.

## Discussion

In this study, occupational and physical therapists screened breast cancer patients postoperatively to enable the early detection of lymphedema, leading to early intervention. As a result, we were able to refer 33 of the 107 patients to a doctor. A total of 23 patients were evaluated during the intervention. These patients received medical attention and early intervention from nurses.

On average, lymphedema occurred seven months postoperatively in this study, with an onset ranging from three to 20 months postoperatively. Following surgery, the incidence of breast cancer-related lymphedema increases from 13.50% after two years, 30.20% after five years, and 41.10% after 10 years [24]. A trend showing an increase in breast cancer-related lymphedema has also been reported during the first year after surgery [25]. Although postoperative breast cancer patients return home after a short hospital stay, this study also found that patients with lymphedema after returning home can be recognized through screening by occupational and physical therapists. In addition, since lymphedema can occur more than a year after discharge from the hospital, healthcare professionals must be involved for an extended period.

In this study, before discharge, patients were provided with education on self-management, including lymphedema. A woman’s ability to recognize symptoms of lymphedema early, seek prompt medical attention, and implement self-management strategies is critical to minimizing its occurrence and progression [26]. However, there are limitations to the ability of patients to detect lymphedema and respond appropriately on their own. In this study, occupational and physical therapists provided periodic lymphedema assessments postoperatively for such patients. As a result, medical professionals were able to detect potential lymphedema in patients at an early stage and refer them.

Lymphedema requires lifelong care because untreated symptoms may worsen and become increasingly difficult to manage as the disease progresses [27]. Early detection and intervention before it becomes severe are crucial for minimizing the development and progression of lymphedema [26]. For the postoperative management of lymphedema, it is necessary to perform evaluations every three months for one to two years after surgery [28,29]. Regular evaluations after discharge from the hospital in this study enabled the early detection of lymphedema. Assessments conducted by occupational and physical therapists, including measurements of upper extremity circumference, were also useful in screening for lymphedema. Therefore, it is essential for healthcare providers to evaluate patients after discharge regularly, and for patients with possible lymphedema to be examined and subsequently treated by a physician.

There was no significant improvement in upper extremity circumference due to our outpatient intervention program, but there was no worsening observed either. There were two patients whose stage progressed, 19 patients who showed no changes, and two patients who showed improvement. In addition, 13% of the cases required intensive drainage due to hospitalization, and 9% of patients underwent LVA. Therefore, our intervention methods showed some effectiveness in preventing the severity of lymphedema. However, since there are limitations to verifying efficacy in the intervention group alone, it will be necessary to establish a control group in addition to the intervention group for comparison in the future.

The limitations of this study are described as follows. First, we did not have a control group, so we cannot verify the causal effect of our intervention. Second, the study was conducted at a single facility, as it was not possible to conduct a study that included multiple facilities. Because the medical personnel are experts in cancer care, the facility’s characteristics may have influenced the results. Future research should consider involving multiple facilities to enhance the study’s validity. Third, the number of patients in this analysis was smaller than the number calculated for the sample size. Fourth, although the degree of lymphedema was evaluated using upper extremity circumference and lymphedema staging, detailed assessments of body water content may be obtained with body composition assessments, a consideration for future research studies.

## Conclusions

Postoperative breast cancer patients are at risk of developing lymphedema after discharge from the hospital. Postoperative assessment by occupational and physical therapists led to the detection of lymphedema at an early stage. As a result, patients were able to prevent the lymphedema from worsening through early treatment, and this intervention method was found to be effective to a certain degree. Postoperative breast cancer patients require continuous evaluation even after discharge from the hospital, and a system for early detection and early treatment of lymphedema is needed.
